# Addressing the deprived: need and access of sexual reproductive health services to street adolescents in Ethiopia. The case of Nekemte town: mixed methods study

**DOI:** 10.1186/s13104-019-4850-7

**Published:** 2019-12-27

**Authors:** Abdo Abazinab Ababor, Desalegn Wirtu Tesso, Melese Chego Cheme

**Affiliations:** 1 Health Office, Buno Bedele Zone, Chora District, Bedele, Oromia Ethiopia; 2 Wollega University, Institute of Health sciences, Nekemte, Ethiopia

**Keywords:** Street adolescents, Nekemte, Sexual reproductive health, Ethiopia

## Abstract

**Objectives:**

Globally, the research knowledge gap exists in the sexual reproductive health (SRH) services of street adolescents. The intensity of the problem is high in settings like Ethiopia, where there are limited access and integration of services. This study aimed at exploring risky sexual behaviors, needs, and barriers of SRH services among street adolescents in Nekemte town. A community-based cross-sectional study design with mixed approaches was used on a sample size of 219 street adolescents. Supplementary qualitative data of 24 in-depth interviews were collected from the street adolescents and SRH service providers. Time-location sampling or venue sampling technique (VDT) was used for a quantitative study. Quantitative data were analyzed by SPSS version 24.0.

**Results:**

About 93% of street adolescents reported difficulty in accessing contraceptives. Behavioral change and sustainable access to SRH services are lacking among street adolescents. The Knowledge gap is more evident in early adolescents (10–13) period than the other classes. In general, street adolescents are deprived of access to SRH services. Mobile and flexible access to contraceptives should be designed targeting street adolescents.

## Introduction

Adolescence is a transition period between childhood to adulthood (10 to 19 years), which is marked by a continuum of physical, mental, behavioral, emotional, and social changes. Streetlife is a global phenomenon and increasing in urban areas of developing countries like Ethiopia. Adolescents on streets are neglected in policy and action to access SRH services [1, 2].

Street adolescents are pushed to street life by complex factors: socioeconomic, political, cultural, and global opportunities and challenges. The intensity of the problem is high in settings like Ethiopia [3–5]. Systemic reviews and studies in Africa show that street children are exposed to street life, mainly due to poverty and abuse. There are more girls on the street than boys [6, 7, 22].

Despite global support and attention, there has been historical neglect of adolescent SRH that exposed the adolescents to risky sexual practice and early parenthood in developing countries [8–13]. As evidence shows, poor adolescents, including street adolescents, are exposed to marginalization in social, political, and economic aspects and face discrimination, stigmatizations, isolations, and violations [14, 15].

Globally, about 13,000,000 women aged 15–19 years give birth annually. Pregnancy and childbirth-related deaths are the primary cause of women’s death in this age group. Furthermore, neonatal mortality of teenage mothers is also higher than adult mothers [2, 13, 16, 17]. In Sub-Saharan Africa, Children and adolescents are among high risk and vulnerable groups for sexual abuse, violence, and HIV/HIDS, and there is a downgrading of vulnerability perception to HIV/AIDS among adolescents [18–25].

Qualitative and quantitative studies also indicate a low level of knowledge of street children on SRH issues, including HIV/AIDs and different forms of violence of abuse to street children, especially girls. As indicated in the studies, street adolescents are engaged in sexual activities early, and they have multiple sexual partners in most of the cases [26–30].

**Fig. 1:**
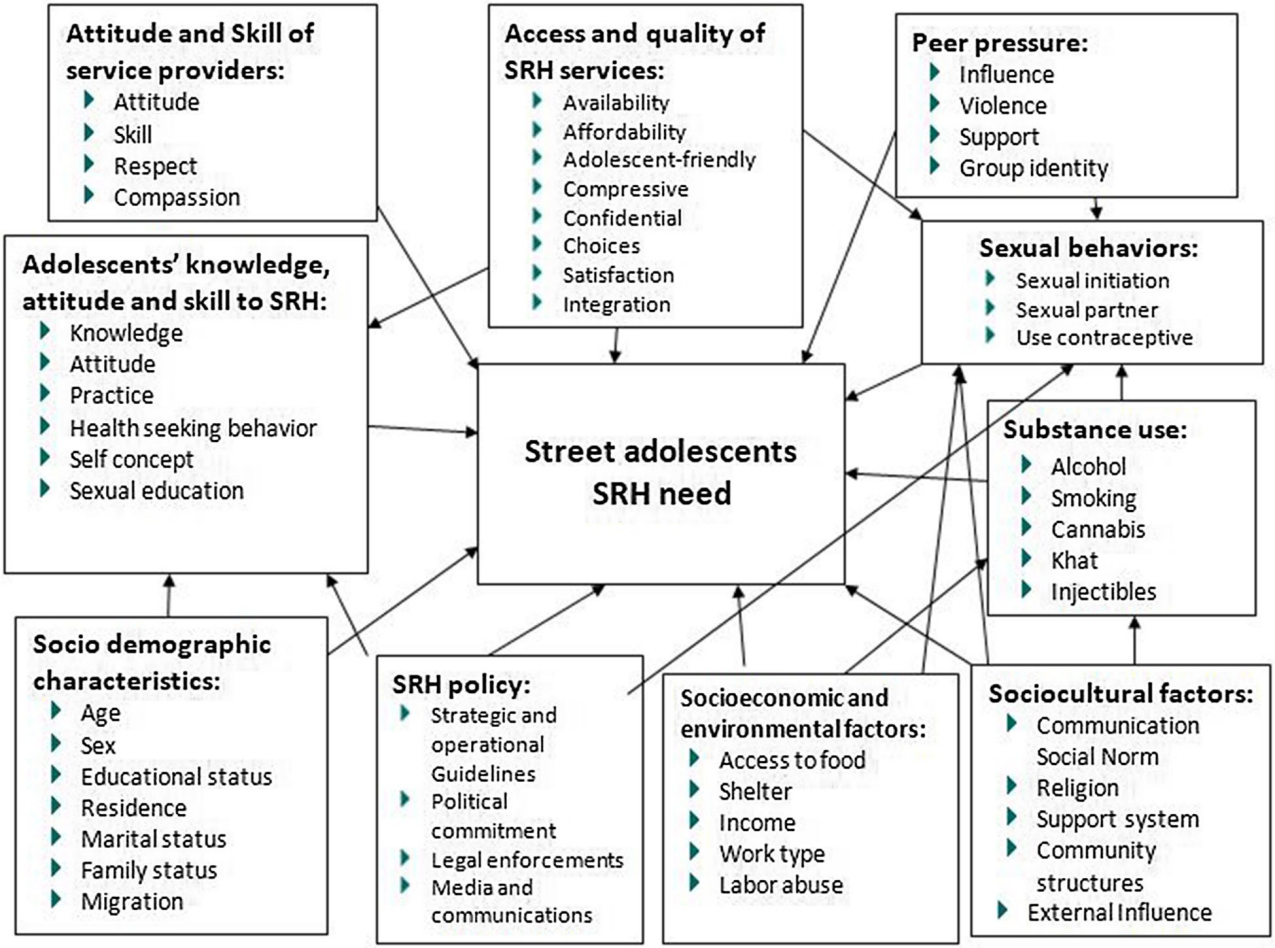
Conceptual framework of the study, developed by the researchers by reviewing literatures, modified by March 17, 2018

In developing countries, a research knowledge gap exists in addressing access to SRH services to populations, particularly the adolescents, despite the increase in demand and challenges. The specific objectives of the study are exploring risky sexual behaviors, SRH needs, and barriers of SRH services utilization among adolescents (10–19 years) in Nekemte town [3, 31]. The conceptual framework of the study is indicated as follows (Fig. 1).

## Main text

### Methods

#### Study setting and study population

The study was conducted from April to June 2018 in Nekemte town, East Wollega Zone, Ethiopia. The town has an estimated total population of 115, 741 in 2016 [32]. The town attracts many adolescents and youths from different urban and rural areas of Ethiopia. The source population for this study was street adolescents aged (10–19 years) who are living or working on the street in Nekemte town.

#### Study design

A community-based cross-sectional study design with mixed methods was used. The study is primarily quantitative, and the qualitative approach is used to accompany the quantitative findings.

#### Sample size determination

The sample size for the quantitative data was determined using a single finite population proportion formula. The assumptions for the sample size (n) calculation were: Proportion (P) of barrier to SRH services among street adolescents in Addis Ababa city in Ethiopia which was 84.7%, confidence level for study 95%, non response rate of 10%, standard error (d) of 5%, and two-sided Z score at 95% confidence interval, 1.96.

$$\therefore n = \frac{{\left( {Z_{/2} } \right)^{2} {\text{P}}(1 - {\text{P}})}}{{{\text{d}}^{2} }} = 235.07$$

Adding a 10% response rate to the initial sample size (n), the total number of employed street adolescents was 235. For supplementary qualitative data, 20 in-depth interviews were proposed initially, and 24 in-depth interviews were conducted from the street adolescents and different SRH service providers.

#### Sampling procedure

A sampling of street adolescents for the quantitative study was conducted by time-location sampling or venue sampling technique, which is recommended for enrolling research participants in special situations like street adolescents in which there is no clear formal structure of the study population to apply the usual probability sampling techniques. The units of the sampling were venue day time units (VDT). Venue day time unit implies the period of some time in a particular venue in a day.

This sampling technique is a probability sampling approach provided that the units are well studied and identified before actual sampling. Individuals included in day time units have an equal chance to be selected in the study. Accordingly, before data collection, some street adolescents and local administrative, religious, and community members were interviewed to identify the day time units and make arrangements. In the study area, 88 venues/locations were identified, and the average number of street children identified in each place for 2 h (VDT) was 12. Based on these the total venues divided by the average number of street adolescents in each VDT gives the number of locations, venues for the study, which are 20 VDTs. Finally, the Street adolescents found in selected VDT were interviewed, for qualitative data participants were purposely selected from street adolescents and reproductive health service providers. The service providers for in-depth interviews were selected based on the information obtained from the Family Guidance Association of Ethiopia Nekemte area coordinator and other key informants about service providers.

#### Data collection procedure

Quantitative data were collected by interviews guided by structured questionnaires after 2 days of training for data collectors. For qualitative data, in-depth interviews were conducted by trained masters level data collectors in the local language, and notes were taken manually with maximum effort.

#### Data processing and analysis

Quantitative data were analyzed using SPSS version 24.0. Descriptive statistics like frequency, percentage, mean, standard deviation were used to describe the findings, and the summary findings were presented by textual, tabular, and graphic presentations. Qualitative data from the in-depth interview were analyzed by thematic analysis approach after translated to English from the local languages, and the responses were coded and sorted to identify themes. The result was presented triangulating and accompanying the quantitative findings. Direct quotes of the respondents were also used.

### Result and discussion

#### Socio-demographic and economic characteristics of street adolescents

About 219 adolescent street adolescents were interviewed, making a 93% response rate. The age range of those adolescents included in this study was between 10 and 19 years, with a mean age of 16.82 (SD ± 1.73) years (Table 1).

**Table 1:** Socio-demographic and socioeconomic characteristic of street adolescent in Nekemte town, April–June 2018

Category	Frequency	Valid Percent
Sex of the street adolescents		
Male	78	35.6
Female	141	64.4
Total	219	100.0
Age group of the street adolescents		
10–13	7	3.2
14–16	62	28.3
17–19	150	68.5
Total	219	100.0
Adolescent engaged in money earning activities		
Yes	172	78.5
No	47	21.5
Total	219	100.0
Type of main money earning activity		
Shoe shining		
Yes	36	16.4
No	183	83.6
Total	219	100.0
Petty trading		
Yes	29	13.2
No	190	87.8
Total	219	100.0
Carrying small items		
Yes	31	14.2
No	188	85.8
Total	219	100.0
Washing cars		
Yes	10	4.6
No	209	95.4
Total	219	100.0
Exchanges money for sex		
Yes	35	16.0
No	184	84.0
Total	219	100.0
Smuggler (transport facilities, selling items)		
Yes	9	4.1
No	210	95.9
Total	219	100.0
Peddling		
Yes	16	7.3
No	203	92.7
Total	219	100.0
Others		
Yes	16	7.3
No	203	92.7
Total	219	100.0
With whom the adolescent live most of the time		
Both parents	20	9.5
Mother	46	21.8
Friends/peers	58	27.5
Alone	87	41.2
Total	211	100.0

#### Substance use behaviors

Different types are substances that are used by street participants. About one-third of the participants use compound or many kinds of substances at a time (Table 2).

**Table 2:** Uses of substance/drug of street adolescent in Nekemte town, September 2018

	Frequency	Percent
Do you use any substance?		
Yes	83	37.9
No	136	62.1
Total	219	100.0
Do you drink alcohol?		
Yes	73	33.3
No	146	66.7
Total	219	100.0
Do you chew chat?		
Yes	79	36.2
No	139	63.8
Total	218	100.0
Why did you decide to use any substance?		
Peer pressure	15	18.1
For practice	1	1.2
For recreation	24	28.9
For time wast	4	4.8
For relief stress/hunger	39	47.0
Total	83	100.0

#### Practice towards SRH issues among adolescents

About 185 (84.5%) know the means of prevention of HIV/STI. Among those 3 (1.4%), 174 (79.5%), 34 (15.5%), and 3 (1.4%) mentioned abstinence, use of a condom, remaining faithful to a partner, and avoiding casual sex, respectively. About 127 (58%) of them reported that they know at least one means of preventing pregnancy. Oral pills, condoms, and injection were the most recognized contraceptive methods that were reported by 95%, 43%, and 93% of the interviewee, respectively. From in-depth interview awareness about un-intended pregnancy, STI/HIV, their transmission and prevention methods are fair, especially for older adolescents. There is a considerable concern to early adolescents (10–13) as this seems to have low decision making power and compression of life matters. It cannot be said that street adolescents do not know SRH issues.

The majority of street adolescents 160 (73.1%) had ever practiced sexual intercourse, 49 (63%) of boys and 111 (79%) of girls. The overall mean age at first sexual initiation was 15.18 years (16.25 years for boys and 14.44 for girls). The main reasons for sexual activity among sexually active Street adolescents were peer pressure 81 (50.6%), and 79 (49.2%) exchange for money. Of those sexually active, the first sexual partner includes causal partner 57 (26%), steady boy/girlfriend 73 (33.3%), and commercial sex worker 16 (7.3%). Sexually active street children were also asked about their sexual experience within the last 12 months. Of those who are currently sexually active, 134 (83.75%) of them reported that they had sexual intercourse with two and more than two partners and the mean number of sexual partners for them was 2.85 (SD ± 0.4) while only 20 (12.5%) have a single sexual partner. Of those sexually active street adolescents, 105 (79.04%) had ever used modern contraceptives, out of which, Condoms 83 (79.04%), Pills 13 (12.3%) injectable 13 (8.12%) were reported to be the most frequently used methods of contraceptives. As reasons mentioned for not using contraceptives include (multiple responses considered): Lack of adequate knowledge 28 (51%), unplanned sex 31 (56%), too far to get contraceptive (not accessible) 32 (58%) and having infrequent sex 19 (35%).

Among the street girls who participated in this study, more than half 80 (57%) reported that they had ever been pregnant, and all 80 (57%) of female respondents said that pregnancies were unwanted. Further, the reasons for pregnancies were accidental 45 (56.25%), unavailability of contraceptive 29 (36.25%), failure of contraceptives 3 (3.75%) was mentioned as the main reasons for the occurrence of unwanted pregnancies.

An in-depth interview with a service provider, FGAE, Nekemte branch claims: “*Trading sex is found to be a serious and growing problem in Nekemte town for street children, particularly amongst street girls. Besides education and service provision, the basic thing is social and financial support”.*

#### Access to SRH service

From who was not seeking SRH care, about 93% reported difficulty in getting contraceptives/condoms. The reasons mentioned for the difficulty were lack of money (89%), too far to get it (86.5%), inconvenience of distribution places (67%), expensive (7.3%), and provider disapproval (15%).

From an In-depth interview, although they use a condom on occasions, it is difficult for adolescents to maintain consistent use of condoms as there are different level barriers. Male, 17 years says, “*Even though they (street adolescents) have enough knowledge on pregnancy, STI/HIV, they don’t stay in safe sexual practice because of peer pressure, negligence, lack of hope, substance abuse, and lack of continuous education on this issue*.”

They have also reported that in terms of access to SRH services, they are disconnected from the existing service stream. A 17-year-old male child suggests, *“….When we visit health facilities, they don’t cooperate well. The workers are usually busy, and you don’t get adequate service for your needs. Most of the street children’s attention is dominated by another issue like cloth, food, and shelter, and they also seek care rarely.*”

Program coordinator of FGAE Nekemte branch states, “*The reality of providing such service (facility*-*based service) is that many street children clients will not attend arranged appointments and may disengage entirely for periods. In these stages, the clients can be most vulnerable, and despite not looking for it, they are often most in need of a sexual health service. It is, therefore, crucial to offering outreach and support to access sexual health services for those children who are most vulnerable and at risk.*” The government health facility director also says, *“Street adolescents had very limited access to reproductive health services. The main reasons were location of the reproductive health facilities and the service providers. The facilities are mostly in residential areas, and the street children mostly operate from Central Business area, therefore crucial to offer outreach and support to access sexual health services”.*

### Conclusion

The main barriers to access local SRH services among Nekemte town street adolescents are lack of information on available services for street adolescents, the behavior of adolescents, inaccessibility adolescent-friendly service. The finding implies that street adolescents are highly deprived and need a particular focus on intervention. Accessing mobile peer-based and friendly services at facilities and in the community should be focused, and rigorous qualitative and quantitative studies should be conducted at a large scale and large sample size to identify the root causes.

## Limitation

The nature of the cross sectional study may not allow the cause-effect relationship and the statistical inference. Social desirability might have affected the responses of street adolescents. Statistical inference is also not reported. Besides, the study should have included the broader views of community, schools, religious institutions, and other relevant bodies. Considering these issues in further research will be helpful.

## Data Availability

Relevant data are available from the corresponding author on a reasonable request.

## Abbreviations

SRH: sexual reproductive health
VDT: venue day time unit
STI: sexually transmitted illnesses
ICT: Information Communication Technology
CSW: commercial sex workers
FGAE: Family Guidance Association of Ethiopia

## References

[1] Borise S, et al. Adolescent sexual and reproductive health toolkit for humanitarian settings, a companion to the inter-agency field manual on reproductive health in humanitarian settings. 2009. https://pt.scribd.com/document/376195634/UNFPA-ASRHtoolkit-english-pdf.

[2] Boakye-Boaten A. An examination of the phenomenon of street children communities in Accra (Ghana). 2006.

[3] Chandramouli V, Svanemyr J, Amin A (2015). Twenty years after international conference on population and development: where are we with adolescent sexual and reproductive health and rights ?. J Adolesc Health.

[4] Kamanu R, Nganga PZ, Muttunga J. Determinants of sexual and reproductive health among street adolescents in Dagoretti District of Nairobi. p. 1–12.

[5] Society T (2014). Sexual and reproductive health care: a position paper of the society for adolescent health and medicine. J Adolesc Health.

[6] Cumber SN, Tsoka-gwegweni JM (2015). The health profile of street children in Africa: a literature review. J Public Health Afr.

[7] Yizengaw SS, Gebiresilus AG. Triggering factors, risky behaviors, and resilience of street children in Gondar City, North West Ethiopia. 2014;2(4).

[8] Morris JL, Rushwan H (2015). Adolescent sexual and reproductive health: the global challenges. Int J Gynecol Obstet.

[9] Salam RA, Faqqah A, Sajjad N (2016). Improving adolescent sexual and reproductive health : a systematic review of potential interventions. J Adolesc Health.

[10] Warenius L (2008). Sexual and reproductive health services for young people in Kenya and Zambia providers’ attitudes and young people’s needs and experiences.

[11] Shaw D (2009). Access to sexual and reproductive health for young people: bridging the disconnect between rights and reality. Int J Gynecol Obstet.

[12] Woan J, Lin J, Auerswald C (2013). The health status of street children and youth in low- and middle-income countries: a systematic review of the literature. J Adolesc Health.

[13] Rushwan H. Adolescent sexual and reproductive health initiative: what do we know about adolescents? Literature review, FIGO.

[14] PAHO. Reaching poor adolescents in situations of vulnerability with sexual and reproductive health. Washington, DC; 2013. https://www.paho.org/hq/index.php?option=com_docman&view=download.

[15] Chase E, Aggleton P, Ingham R, Aggleton P (2006). Meeting the sexual health needs of young people living on the street. Promoting young people’s sexual health: international perspectives.

[16] Igras SM, Macieira M, Murphy E, Lundgren R (2014). Investing in very young adolescents’ sexual and reproductive health. Glob Public Health.

[17] Chandramouli V, Camacho AV, Michaud P (2013). WHO guidelines on preventing early pregnancy and poor reproductive outcomes among adolescents in developing countries. J Adolesc Health.

[18] Karki S, et al. Risks and vulnerability to HIV, STIs and AIDS among street children in Nepal: public health approach. Post Doctoral thesis, University of Huddersfield; 2013. http://eprints.hud.ac.uk/id/eprint/21282/.

[19] Habtamu Demelash, Adamu Addisie (2013). Assessment of Sexual and Reproductive Health Status of Street Children in Addis Ababa. Journal of Sexually Transmitted Diseases.

[20] East, Central, and Southern African Health Community, child sexual abuse in sub-Saharan Africa. Literature review; 2011. https://www.svri.org/sites/default/files.

[21] Kumi-Kyereme A, Awusabo-Asare K, Biddlecom A. Adolescents’ sexual and reproductive health: qualitative evidence from Ghana; 2007. https://www.guttmacher.org/pubs/2007/08/31/or30.pdf.

[22] Bam K. Scenario of adolescent sexual and reproductive health with opportunities for information communication and technology use in selected South Asian Countries. iMedPub J. Access to ICT and Challenges of ASRH program. 2015;1–7.

[23] Cumber S. Pattern and practice of psychoactive substance abuse and risky behaviors among street children in Cameroon. 2016;10(3).

[24] Wittenberg J (2007). Protecting the next generation in Malawi: new evidence on adolescent sexual and reproductive health needs.

[25] Publications MJ, Berhane T, Assefa B, Birhan N (2014). Reproductive health behavior of street youth and associated factors in Gondar City, Northwest Ethiopia. Int J Med Biomed Res.

[26] Uddin MJ, Sarma H, Wahed T (2014). The vulnerability of Bangladeshi street-children to HIV/AIDS: a qualitative study. BMC Public Health..

[27] Shaikh BT, Rahim ST (2006). Assessing knowledge, exploring needs: a reproductive health survey of adolescents and young adults in Pakistan. Eur J Contracept Reprod Health Care.

[28] Wachira J, Kamanda A, Embleton L, Naanyu V, Winston S, Ayuku D (2015). Initiation to street life: a qualitative examination of the physical, social, and psychological practices in becoming an accepted member of the street youth community in Western Kenya. BMC Public Health.

[29] Habtemariam K. Knowledge, attitude and practice of modern contraceptives among street girls of bole sub-city, Addis Ababa. http://www.localhost:80/xmlui/handle/123456789/1976.

[30] Kayembe PK, Mapatano MA, Fatuma AB (2008). Knowledge of HIV, sexual behaviors and correlates of risky sex among street children in Kinshasa, Democratic Republic Of Congo, East African. J Public Health.

[31] Hughes Jane, McCauley Ann P. (1998). Improving the Fit: Adolescents' Needs and Future Programs for Sexual and Reproductive Health in Developing Countries. Studies in Family Planning.

[32] Population estimation of Nekemte for 2016, based on data on List of cities and towns in Ethiopia, Wikipedia, the free encyclopedia. https://en.wikipedia.org/wiki/Nekemte.

